# Application of the “risk of ambulatory disability” (RoAD) score in a “real‐world” single‐center multiple sclerosis cohort

**DOI:** 10.1111/cns.13806

**Published:** 2022-01-21

**Authors:** Maximilian Pistor, Helly Hammer, Anke Salmen, Robert Hoepner, Christoph Friedli

**Affiliations:** ^1^ Department of Neurology Inselspital Bern University Hospital, and University of Bern Bern Switzerland

**Keywords:** disability, immunotherapy, MS, prediction score

## Abstract

Survival analysis of reaching EDSS ≥4.0 based on RoAD score ≥4 (dashed line) and <4 (solid line) by Cox regression analysis. (A) Unadjusted regression analysis. (B) Regression controlled for sex and immunotherapy groups, and the trajectory of treatment changes during follow‐up.

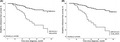

## CONFLICTS OF INTEREST

Pistor M declares no conflicts of interest. Hammer H received research support and travel grants within the last 5 years from Biogen, Merck, Roche, and BMS. Salmen A received speaker honoraria and/or travel compensation for activities with Almirall Hermal GmbH, Biogen, Merck, Novartis, Roche, and Sanofi Genzyme and research support by the Swiss MS Society. Hoepner R received research and travel grants from Roche, Novartis and Biogen Idec and speaker honoraria from Biogen, Novartis, Merck, Celgene, and Almirall, not related to this work. Friedli C received speaker honoraria and/or travel compensation for activities with Biogen, Sanofi Genzyme, Novartis, and Merck, not related to this work.

## AUTHOR CONTRIBUTIONs

Pistor M was responsible for conceptualization, investigation, formal analysis, obtained and analyzed the data and revised the manuscript. Hammer H and Salmen A interpreted the data and revised the manuscript. Hoepner R was responsible for conceptualization, methodology, analyzed the data, drafted and revised the manuscript. Friedli C was responsible for conceptualization, reviewed the literature, interpreted the data, prepared the original draft and revised the manuscript.

Dear Editor,

Recently, a study predicting the risk of ambulatory disability in multiple sclerosis (MS) over a period of 10 years based on demographic, clinical baseline prognostic factors and 1‐year predictors of treatment response was published.^1^ The investigated cohort consisted of patients with a relapsing‐remitting (RR) disease course with an initial expanded disability status scale (EDSS) <4.0, an initial treatment of any formulation of interferon beta (IFNB) or glatiramer acetate (GA), 6‐monthly EDSS assessments and brain magnetic resonance imaging (MRI) scans at baseline and after 1 year of treatment. The study revealed, that a score based on readily available variables at baseline and 1‐year follow‐up is able to predict ambulatory disability over a period of 10 years. Another study^2^ comparing different treatment response scoring systems such as risk of ambulatory disability (RoAD) score, Rio score, and modified Magnetic resonance imaging in MS (MAGNIMS) score was recently published, demonstrating a good sensitivity of RoAD score >3 to predict the risk of reaching EDSS 4 and 6 in cohort treated with an injectable therapy (IFNB and GA). Prediction of an unfavorable course of MS early during the disease is of utmost importance in clinical practice,^3^, ^4^ especially in the light of the rapidly evolving therapy landscape. Therefore, we aimed to independently investigate the “Risk of Ambulatory Disability” (RoAD) score in a clinical university care setting for people with MS in Switzerland, also including a relevant proportion of recently approved immunotherapies.

We conducted a retrospective, monocentric analysis on an independent real‐world cohort of MS patients treated at University Hospital Bern diagnosed 2005–2017. Patients with a diagnosis of RRMS,^5^ treated with any MS immunotherapy and excluding patients not receiving an immunotherapy, an EDSS <4.0 at baseline, defined as the first diagnosis of MS, an EDSS<4 after 1 year of follow‐up, regular clinical follow‐up visits with assessment of EDSS at least each year (+/‐ 3 months) as well as routine cerebral MRI at baseline and after 12 months (+/‐ 3 months) were identified by chart review and followed until August 2020. Survival analyses on the outcomes of interest (primary outcome EDSS ≥4.0 and secondary outcome EDSS ≥6.0) were performed in a time‐to‐event fashion by Cox regression analysis applying the RoAD score dichotomized (<4 vs. ≥4) as independent variable. Since various types of immunotherapies have been used (Table 1), we controlled for immunotherapies during the first year of observation by classifying them according to the European Medical Agency (EMA) (figure legend 1). Since changes in immunotherapies over the follow‐up period might influence outcomes, we controlled for the trajectory of changes (no change of immunotherapy, escalation from EMA 1st to 2nd line, change within the EMA 1st or 2nd line therapies (“lateral/horizontal switch”), de‐escalation; distribution see Table 1). IBM SPSS Statistics 25 (USA, 2017) was used. Hazard ratios (HR) and other metrics were displayed with the 95% confidence intervals (95% CI); *p* < 0.05 was considered significant. An anonymized data set will be handed over to any researcher upon reasonable request via the corresponding author.

**TABLE 1:** Showing baseline and 1st year follow‐up characteristics as well as the immunotherapy during the first year after diagnosis

Cohort (*n* = 194)	Mean/Proportion	95% confidence interval	Range
Sex (female, *n* [%])	114/194 (58.8%)	.	.
Age at diagnosis (years)	34.9	33.3–36.5	15–69
EDSS score at baseline	1.8	1.6–1.9	0–3.5
Time between first symptoms and diagnosis (month)	22.3	15.7–28.8	0–358
Observation time (month)	78.3	72.6–84.0	20–204
Number of relapses in the 1st year of diagnosis	0.4	0.3–0. 5	0–4
Number of Gadolinium‐enhancing lesions on the MRI 12 months (+/‐ 3 months)	0.7	0.3–1.1	0–30
Number of T2‐ hyperintense lesions on the MRI 12 months (+/‐ 3 months)	2.2	1.6–2.7	0–29
1st year immunotherapy:		.	.
Interferon beta	89/163 (54.6%)	.	.
Glatiramer acetate	14/163 (8.6%)	.	.
Fingolimod	21/163 (12.9%)	.	.
Dimethyl fumarate	25/163 (15.3%)	.	.
Teriflunomide	1/163 (0.6%)	.	.
Natalizumab	12/163 (7.4%)	.	.
Ocrelizumab	1/163 (0.6%)	.	.
Immunotherapies changes (*n*, [%])			
	Not changed compared to 1st immunotherapy	59/163 (36.2%)	.
	Treatment intensification	58/163 (35.6%)	.
	“Lateral shift”	43/163 (25.8%)	.
	Treatment de‐escalation	4/163 (2.5%)	.

Note

Changes in immunotherapies based on the EMA 1st and 2nd line therapies are displayed, “lateral shift” represents a change in immunotherapy within EMA 1st or 2nd line therapies.

Abbreviation: EDSS, extended disability status scale, EMA, European Medical Agency.

This study was approved by the responsible cantonal ethics committee (registration no. KEK‐BE ethic vote: BE 2017–01369). Because of the retrospective nature of the analysis with pseudonymized patient data, separate informed consent was waived by the committee. This corresponds to the local legislation. For patients seen after the introduction of the general consent (February 2015), the presence of the patients' consent was checked before inclusion in the analysis.

**FIGURE 1:**
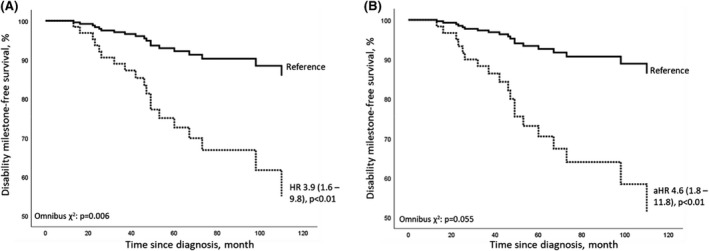
Survival analysis of reaching EDSS ≥4.0 based on RoAD score ≥4 (dashed line) and <4 (solid line) by Cox regression analysis. (a)HR displayed, followed by 95% confidence intervals. Model fitness indicated by Omnibus *χ*2. (A) Unadjusted regression analysis on RoAD score without controlling for sex and immunotherapy groups. (B) Regression controlled for sex (HR 1.5, 0.7–3.1, *p* > 0.05) and immunotherapy groups (“EMA 1st line”: interferon beta, glatiramer acetate, dimethyl fumarate, and teriflunomide; “EMA 2nd line”: fingolimod, natalizumab, and ocrelizumab) and trajectory of follow‐up treatment changes. Immunotherapy (based on EMA 1st or 2nd line) and the trajectory of treatment changes during follow‐up did not significantly influence the outcome (HR 1.06 [0.3–3–8; *p* = 0.92] and HR 0.9 [0.5–1.7; *p* = 0.81]). Abbreviations: EMA: European Medical Agency, (a) HR: (adjusted) hazard ratio, RoAD score: risk of ambulatory disability score

We analyzed data of 163 RRMS patients treated at our neuroimmunological outpatient department of the Inselspital, University Hospital Bern, a tertiary neurological center in Switzerland. The mean age at diagnosis of our cohort was 34 years (95% confidence intervals [95%‐CI]: 32.3–35.7) with 60% being female. Most patients were treated with interferon beta (*n* = 89, 54.6%), dimethyl fumarate (*n* = 25, 15.3%), or fingolimod (*n* = 21, 12.9%) as initial MS therapy (Table 1). During follow‐up, 36.2% of our cohort had no change in their initial treatment, 35.6% had an intensified treatment (defined by EMA 1st and 2nd line therapies), 25.8% changed within EMA 1st or 2nd line therapies, and 2.5% had a treatment de‐escalation. Over the whole observation period, 19 of 163 patients (11.7%) reached an EDSS ≥4.0 after 70.7 months (mean, 95%‐CI: 64.8–76.6) and 4/163 patients (2.5%) reached EDSS 6.0. The distribution of RoAD scores in our cohort is shown in Figure S1. Stratifying by RoAD score <4 (*n* = 134/163) vs. ≥4 (*n* = 29/163) demonstrated a HR of 3.9 of reaching EDSS ≥4.0 (95% CI 1.6–9.8, *p* < 0.01). After adjusting the Cox regression analysis for sex, 1st year immunotherapy and the direction of treatment changes we observed an HR of 4.6 of reaching EDSS ≥4.0 (95% CI 1.8–11.8, *p* < 0.01; see Figure 1). Sensitivity and specificity of the RoAD Score cutoff ≥4 was 42.1% (95% CI 21.1–66.0) and 85.4% (95% CI 78.3–90.5), respectively. The HR of reaching EDSS ≥6.0 of patients with a RoAD score ≥4 was 4.3 (95% CI 0.6–30.7) compared to patients with a RoAD score <4 (*p* > 0.05), not reaching statistical significance after correction for immunotherapy, treatment changes, and sex (HR 6.7; 95% CI 0.7–59.4, *p* = 0.09, Figure S2). A subgroup analysis based on injectable‐treated patients (as described by Gasperini et al.^1^ and Rio et al.^2^) was additionally performed (103/163 patients, Figure S3). The HR of reaching EDSS ≥4 stratified by a RoAD score of ≥4 was 4.0 (1.5–10.7; *p* < 0.01), the adjusted HR 4.6 (1.6–13.1; *p* < 0.01). Concerning the non‐injectable‐treated patients (60/163), the HR (3; 0.3–33.2) and adjusted HR (7.2; 0.4–115) did not meet the pre‐defined level of significance (*p* > 0.05).

In our data set, we could validate the recent findings that proposed the RoAD score as a useful tool to predict individual prognosis of disability progression in patients with RRMS early in the course of the disease.^1^ We found that this score using readily available variables at start of treatment, such as age, time since first MS symptom and EDSS score, and variables after 1 year of treatment, including the number of relapses, number of gadolinium‐enhancing lesions, and number of new T2 lesions, are able to predict the disability milestone of an EDSS ≥4.0 in our single‐center real‐world cohort with a high degree of specificity, however, with only moderate sensitivity. In contrast to the initial studies, we included patients treated not only with injectables but also a relevant proportion of patients receiving newer MS immunotherapies (36.8%) and patients with changes in their treatment during the follow‐up (intensification, “lateral/horizontal switch”). This cohort therefore reflects the common treatment landscape and practice, representing a real‐world cohort. Despite a less standardized research setting, with follow‐up examinations including EDSS assessments every 6–12 months during clinical routine, the RoAD score cutoff ≥4 points still remains predictive for reaching the EDSS score milestone of ≥4.0. This underpins the clinical utility of the easy to calculate RoAD score. A major shortcoming of the study in our setting is that the EDSS score milestone of 6.0 was reached only in a low number of patients, which negatively impacts statistical analysis in this regard and might explain why our analysis missed 95% significance levels. The shorter follow‐up of our cohort compared to the original studies (mean 6.3 years vs. 10 years^1^, ^2^) is an additional contributor as we cannot rule out that more patients would have reached an EDSS of ≥6.0 if we have had a longer follow‐up. Due to the more heterogeneous treatment landscape in our cohort, our analysis was controlled for immunotherapy and, if immunotherapies were intensified, de‐escalated or remained unchanged, since these parameters might have an influence on the risk of disability accrual. Nevertheless, the RoAD score, which includes parameters of disease activity such as contrast‐enhancing lesions in the first year, remains predictive of future disability, underlining the importance of the treatment response within the first year for future disability development. Comparing sensitivity and specificity of RoAD score in our cohort with the prior studies demonstrates a lower sensitivity (41 vs. 61%^2^ and 65%^1^) but comparable specificity (85.4% vs. 77%^2^ and 86%^1^). The reasons for these findings are elusive but might be in parts due to the longer follow‐up and less heterogenic treatment landscape in the original studies compared to our observation. In the future, the RoAD score might be extended including novel biomarkers such as cerebrospinal fluid (CSF) parameters,^6^, ^7^ MRI atrophy monitoring,^8^, ^9^ and neurofilament light chain serum concentrations.^10^ Additional clinical variables, such as early involvement of pyramidal system, bladder problems, or fatigue, might bear prognostic potential.^11^ Finally, our study stresses the importance to test and validate developed prognostication scores in real‐world cohorts outside of clinical studies in independent cohorts.

## Supporting information

Fig S1

Fig S2

Fig S3

## Data Availability

Data and materials are available upon reasonable request via the corresponding author.
