# Supplementary figures and images for: Application of the “risk of ambulatory disability” (RoAD) score in a “real‐world” single‐center multiple sclerosis cohort
Source: CNS Neurosci Ther. 2022 Jan 21;28(5):792–5. doi: 10.1111/cns.13806 (PMC8981428; doi:10.1111/cns.13806)

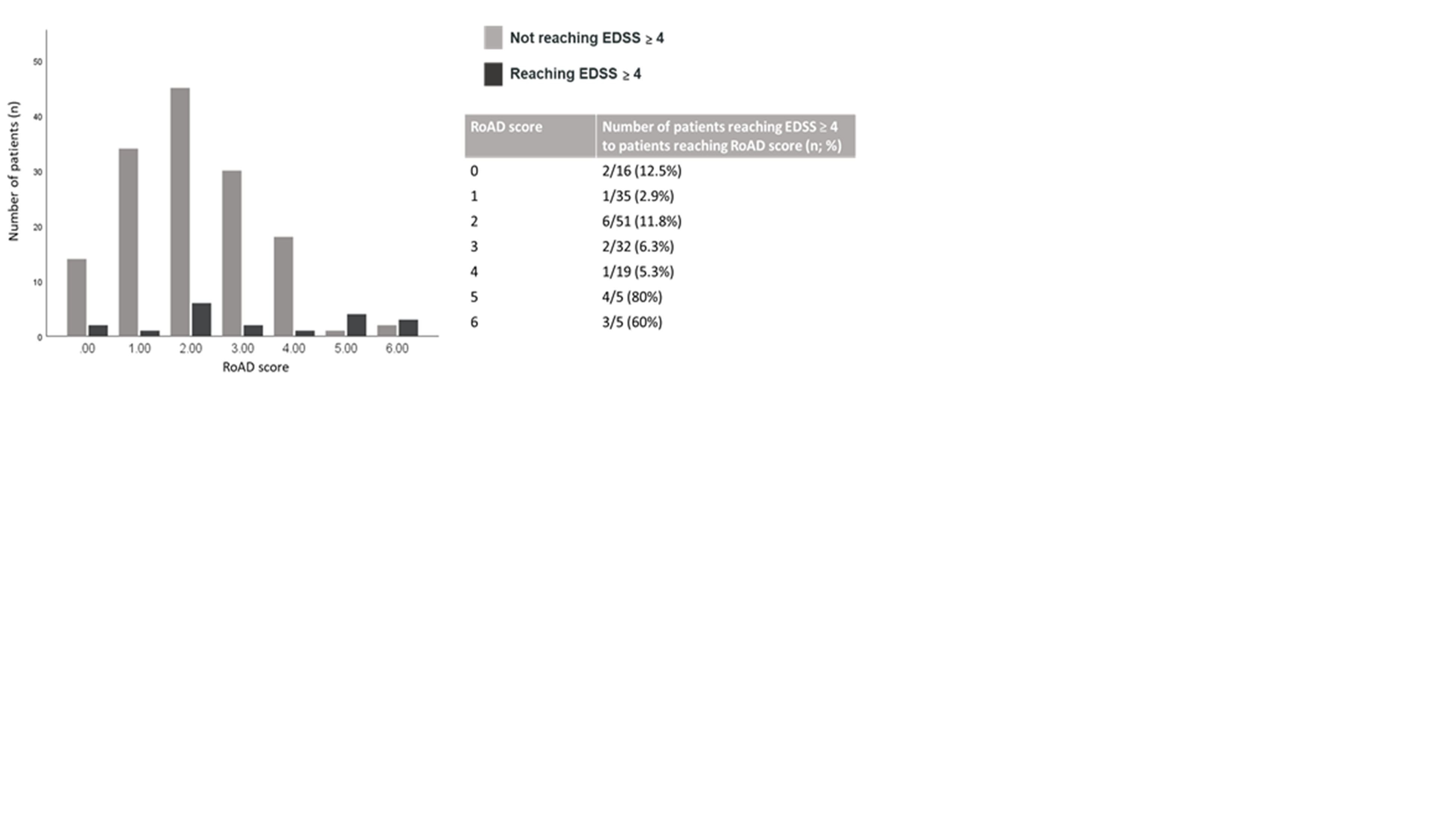

Supplement: Supplementary file 1 — Fig S1 [file CNS-28-792-s003.tif]

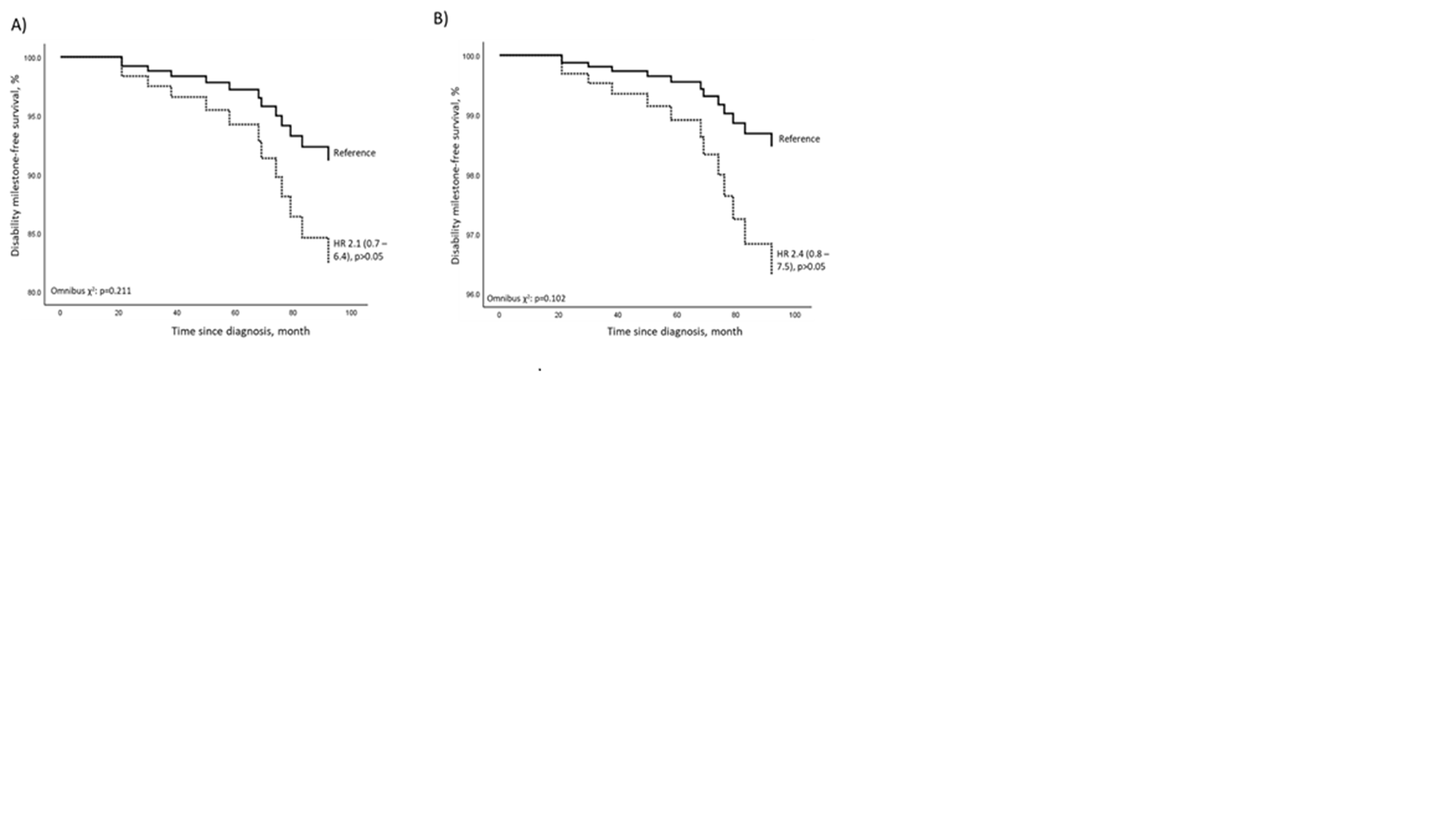

Supplement: Supplementary file 2 — Fig S2 [file CNS-28-792-s002.tif]

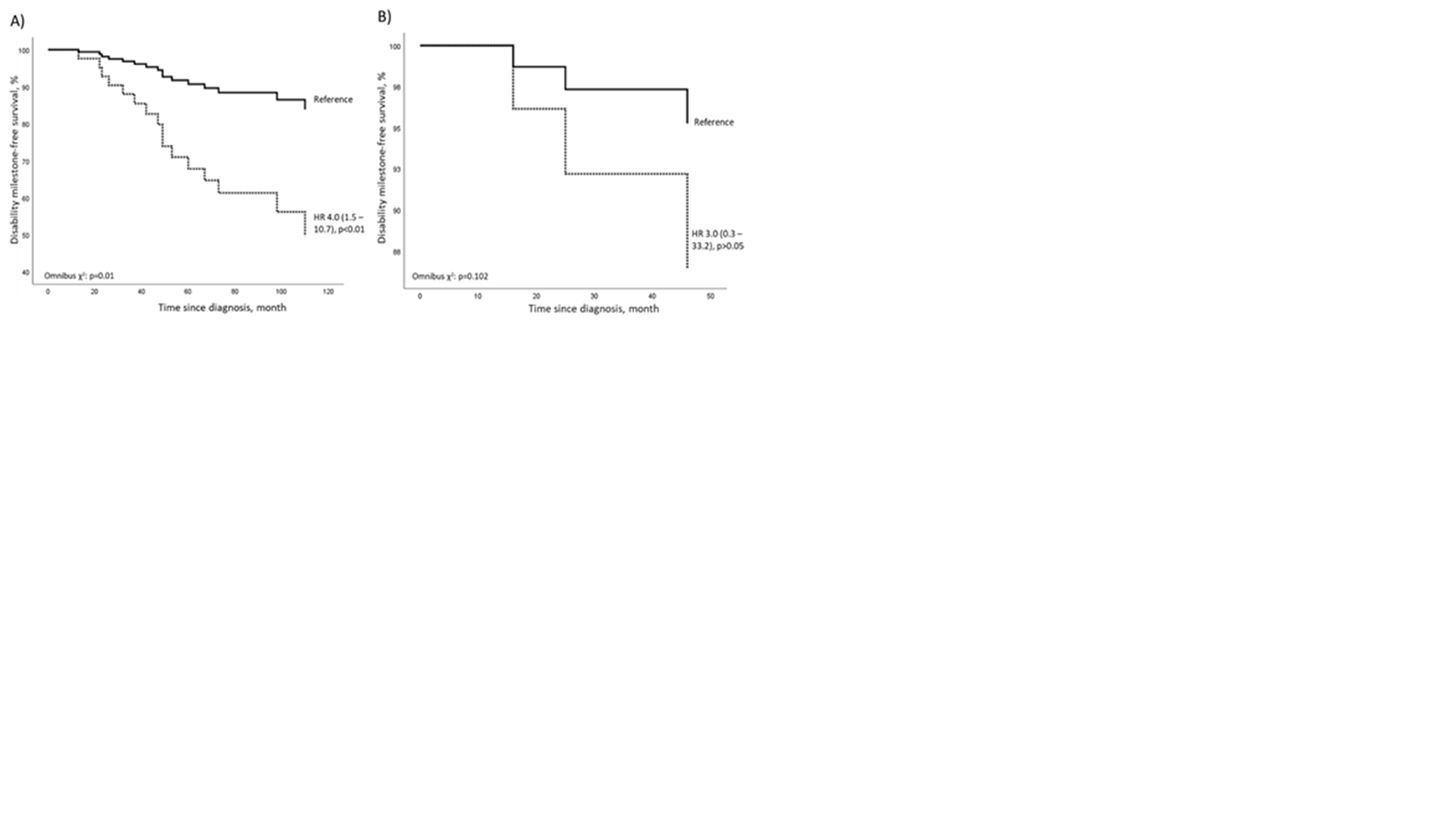

Supplement: Supplementary file 3 — Fig S3 [file CNS-28-792-s001.tif]
